# A biopsy-based Immunoscore in patients with treatment-naïve resectable gastric cancer

**DOI:** 10.1177/17588359241287747

**Published:** 2024-10-21

**Authors:** Tanya T. D. Soeratram, Isis Beentjes, Jacqueline M. P. Egthuijsen, Aart Mookhoek, Marilyne M. Lange, Elma Meershoek-Klein Kranenbarg, Henk H. Hartgrink, Cornelis J. H. van de Velde, Bauke Ylstra, Hanneke W. M. van Laarhoven, Nicole C. T. van Grieken

**Affiliations:** Department of Pathology, Amsterdam UMC location VUmc, Amsterdam, Vrije Universiteit Amsterdam, Amsterdam, The Netherlands; Cancer Center Amsterdam, Cancer Biology and Immunology, Amsterdam, The Netherlands; Department of Pathology, Amsterdam UMC location VUmc, Amsterdam, Vrije Universiteit Amsterdam, Amsterdam, The Netherlands; Cancer Center Amsterdam, Cancer Biology and Immunology, Amsterdam, The Netherlands; Department of Pathology, Amsterdam UMC location VUmc, Amsterdam, Vrije Universiteit Amsterdam, Amsterdam, The Netherlands; Cancer Center Amsterdam, Cancer Biology and Immunology, Amsterdam, The Netherlands; Institute of Tissue Medicine and Pathology, University of Bern, Bern, Switzerland; Department of Pathology, Amsterdam UMC location VUmc Amsterdam, Amsterdam, The Netherlands; Department of Surgery, Leiden University Medical Center, Leiden, The Netherlands; Department of Surgery, Leiden University Medical Center, Leiden, The Netherlands; Department of Surgery, Leiden University Medical Center, Leiden, The Netherlands; Department of Pathology, Amsterdam UMC location VUmc, Amsterdam, Vrije Universiteit Amsterdam, Amsterdam, The Netherlands; Cancer Center Amsterdam, Cancer Biology and Immunology, Amsterdam, The Netherlands; Cancer Center Amsterdam, Cancer Biology and Immunology, Amsterdam, The Netherlands; Department of Medical Oncology, Amsterdam UMC location University of Amsterdam, Amsterdam, The Netherlands; Department of Pathology, Amsterdam UMC location VUmc, Amsterdam, Vrije Universiteit Amsterdam, De Boelelaan 1117, 1081 HV, Amsterdam, The Netherlands; Cancer Center Amsterdam, Cancer Biology and Immunology, Amsterdam, The Netherlands

**Keywords:** biopsy, gastric cancer, prognostic marker, tumor-infiltrating lymphocytes, tumor microenvironment

## Abstract

**Background:**

The prognostic significance of T-cell densities in gastric cancer (GC) was previously demonstrated in surgical resection specimens. For prognosis or response prediction, it is preferable to identify biomarkers in pre-treatment biopsies; yet, its representativeness of the tumor immune microenvironment is unclear.

**Objectives:**

This study aimed to evaluate the concordance and prognostic value of T-cell densities in paired biopsies and resections.

**Methods:**

Paired diagnostic biopsies and surgical resections were available for 131 patients with resectable GC who were treated with surgery alone in the D1/D2 trial. T-cell markers such as CD3, CD45RO, CD8, FOXP3, and Granzyme B were assessed by immunohistochemistry and digitally quantified. Tumors were categorized into high and low subgroups for each marker. The concordance between biopsies and resections was determined for each marker with Cohen’s κ. To determine the prognostic value of T cells in biopsies, Cox regression was performed.

**Results:**

The concordance of T-cell high and low tumors was moderate for CD8 (κ = 0.58) and weak for other markers (κ < 0.3). CD8 and FOXP3 densities in biopsies were significantly associated with cancer-specific survival. Multivariable analysis showed that an Immunoscore incorporating CD8 and FOXP3 served as an independent prognostic marker (low vs high: hazard ratio 3.40, 95% confidence interval: 1.27–9.10; *p* = 0.015).

**Conclusion:**

Although the concordance in T-cell densities between biopsy and resection specimens is modest, a biopsy-based Immunoscore identified distinct biological subgroups with prognostic potential. To fully evaluate the prognostic performance of this biopsy Immunoscore, additional studies are warranted.

## Graphical abstract

**Graphical abstract:**
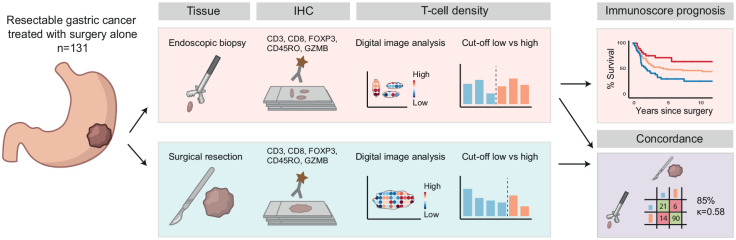

## Introduction

Gastric cancer (GC) has a poor prognosis, particularly since the majority of patients present at an advanced stage. GC ranks as the third most common cause of cancer-related death in men and fifth in women.^1^ In Western countries, perioperative chemotherapy has become the standard treatment for resectable GC. Despite the notable advancements of this treatment approach, the 5-year survival rates of resectable GC remain limited (reaching approximately 40% compared to 20% with surgery alone).^2,3^ Given the biological and clinical heterogeneity of GC, the challenge lies in the classification of patients in different prognostic risk groups which could also impact treatment decisions. To this end, the identification of prognostic biomarkers is imperative and should ideally be determined before treatment commencement.

Tumor-infiltrating T cells play an important role in tumor progression^4^; a high density of tumor-infiltrating T cells has been associated with improved prognosis.^5,6^ We showed in a previous study that CD3^+^, CD45RO^+^, CD8^+^, FOXP3^+^, and Granzyme B^+^ T cells were significantly associated with cancer-specific survival.^7^ T-cell densities also have potential as a predictive marker for response to adjuvant chemotherapy.^8,9^ In these studies,^6–9^ T cells were assessed using tissue sections of surgical resection specimens of treatment-naïve patients. However, the tumor immune microenvironment in surgical resections could be influenced by the pretreatment^10^ given in Western countries, which may lead to different classifications for these patients. Pretreatment endoscopic biopsies are routinely taken for diagnostic evaluation and could serve as substitute tissue specimens. Moreover, in cases where surgery is not indicated, endoscopic biopsies serve as the only tissue specimens available. However, it is not clear how suitable biopsies are for T-cell assessment.

Compared to surgical resections, biopsies have limitations due to their smaller size and hence representativeness for the entire tumor. This concern is furthermore supported by our previous findings using surgical resection specimens demonstrating heterogeneous T-cell distribution with prognostic value depending on T-cell location.^7^ CD8^+^ T-cell densities in the invasive margin showed a stronger association with survival compared to the tumor center.^7,11^ Since endoscopic forceps biopsies are taken from the tumor surface and do not reach the invasive margin, this will have an impact on the tumor representativeness and hence impact on the value of the T-cell markers that were identified in resection specimens. Moreover, immune cells present at the tumor surface could also be associated with an inflammatory response to mucosal damage,^12^ and therefore not related to cancer-specific survival (CSS).

Studies investigating the T-cell density in biopsies and its representativeness for the tumor are currently lacking for GC. This study aims to evaluate whether the T-cell densities determined in GC biopsies are concordant with the density in paired resections and to explore the prognostic potential of biopsy-based T-cell density.

## Methods

### Patients

Patients with resectable gastric adenocarcinoma underwent gastrectomy with D1 lymphadenectomy or D2 lymphadenectomy without perioperative treatments in the randomized nationwide Dutch D1/D2 trial between 1989 and 1993, with a median follow-up time of 15.2 years.^13^ Formalin-fixed and paraffin-embedded (FFPE) tissue blocks of 251 surgical resections were obtained previously.^7^ For this retrospective study, FFPE tumor blocks of diagnostic biopsies from the same 251 patients were requested and obtained via the Dutch National Tissuebank Portal.^14^ Clinicopathological parameters including molecular subgroups were reported previously.^15,16^ For this study, we only included clinicopathological parameters that were known at the time the biopsy was taken or that were determined on biopsy material. However, we did not include the cT and cN stages since these are less reliable than the pT and pN stages. The study was conducted in accordance with the Declaration of Helsinki and the Dutch Code of Conduct for Health Research. The reporting of this study conforms to the REMARK EQUATOR guideline^17^ (Supplemental Document 1).

### Quantification of T cells

Immunohistochemical staining of CD3 (general T-cell marker), CD45RO (memory T-cell marker), CD8 (cytotoxic T-cell marker), FOXP3 (regulatory T-cell marker), and Granzyme B (cytotoxic enzyme) was performed in serial sections of surgical resection specimens in the previous study.^7^ Positively stained T-cell densities were assessed in cells/mm^2^ with the digital image analysis software QuPath. For the biopsies, the same workflow for immunohistochemistry and digital image analysis described in this previous study was used. Deviations included extra hematoxylin and eosin (H&E) staining on the last serial section. Both the first and last H&E sections were evaluated on the presence of tumor cells and the Lauren histological classification by the pathologist N.C.T.G. In the previous study that included the tumor resections, the “tumor center” and “invasive margin” were manually annotated and divided into tiles, but the “tumor surface” was not analyzed separately. For this study, the “tumor surface” of the resection was analyzed by selecting the tiles 1–2 mm from the mucosal edge. In the digital image slides of the biopsies, the tumor area was manually annotated without further classification in the tumor center or invasive margin.

### Statistical analysis

A general comparison of the 131 paired biopsy and resection T-cell densities was performed with the paired Wilcoxon rank-sum test as T-cell densities were not normally distributed. All 149 biopsies were dichotomized in T-cell density high versus low subgroups according to the same method as for the resections, with cutoffs calculated using the maximized log-rank statistic of CSS. CSS was defined as the time from surgery until death due to GC. The concordance of T-cell high and low categories in the 131 paired biopsy and resection specimens was calculated with percentage agreement and Cohen’s κ. Kaplan–Meier and univariable Cox proportional hazards regression were performed to determine the prognostic value of the T-cell and clinical parameters. A multivariable analysis was performed that included the significant T-cell markers from the univariable Cox regression in addition to sex, age, Lauren subtype, microsatellite instability (MSI) status, and Epstein-Barr virus (EBV) status. All tests were two-sided and a cutoff of 0.05 was used for statistical significance. Data processing and statistical analysis were performed in R version 4.0.3.^18^ using packages such as data.table,^19^ survival,^20^ survminer,^21^ irr,^22^ and ggplot2.^23^

## Results

Archival FFPE diagnostic biopsies were retrieved for 131 of the 251 D1/D2 trial patients for which T-cell densities were previously assessed using resection specimens, plus 18 non-matched biopsies (Supplemental Figure S1). The patient characteristics of this subset of patients are depicted in Supplemental Table 1.

**Figure 1.**
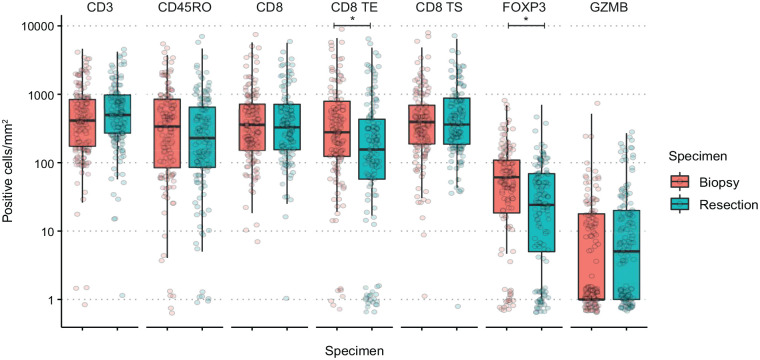
Median T-cell densities in paired biopsy and resection specimens. The median T-cell density value of all tiles in the tumor section (cells/mm^2^) of the biopsy and resection specimens. Each dot represents one tumor sample. The boxplots depict the median and interquartile range of all tumor samples. The density values were offset by 1 before the log transformation. The statistical difference between biopsy and resection was determined by the Wilcoxon ranked paired sum test before log transformation. Statistically significant differences are indicated by*. TE, tumor epithelium; TS, tumor stroma.

The T-cell densities were compared between all paired biopsies and resections using the Wilcoxon-ranked paired sum test, without multiple testing corrections (Figure 1). FOXP3^+^ median T-cell density was significantly higher in the biopsies compared to resections (*p* < 0.001). However, this disparity in FOXP3^+^ T cells was found to be significant solely in intestinal-type tumors and not in diffuse-type tumors (Supplemental Figure S2). Overall, no significant difference in CD8^+^ T-cell density was found between biopsies and resection specimens. However, when examining CD8^+^ T cells based on their intraepithelial or stromal location, a significantly higher number of intraepithelial CD8^+^ T cells were detected in the biopsies (*p* < 0.001). CD3, CD45RO, and Granzyme B^+^ T-cell density were not significantly different between biopsies and resections.

The T-cell densities categorized into high and low subgroups were compared for biopsy versus resections and showed a moderate concordance for CD8^+^ T cells (κ = 0.58), weak for CD45RO and Granzyme B (κ < 0.3), and minimal for CD3^+^ and FOXP3^+^ T cells (κ < 0.2) (Table 1). Distinct levels of concordance were observed when tumors were analyzed according to the Lauren classification separately. The κ value was higher in intestinal-type tumors for CD3, CD8, and Granzyme B, compared to the diffuse-type tumors. For FOXP3, the κ value was lower in intestinal-type tumors compared to diffuse-type tumors (Supplemental Table 2).

**Table 1.** Concordance of high and low T-cell density subgroups in paired biopsies and resections.

Resection	Biopsy				
	High	Low	Concordance (%)	Cohen’s κ	*p*-Value
CD3					
High	20	41	58.8	0.146	0.062
Low	13	57			
CD45RO					
High	14	17	74	0.282	0.001
Low	17	83			
CD8					
High	21	6	84.7	0.58	<0.001
Low	14	90			
CD8 TE					
High	21	3	74.8	0.414	<0.001
Low	30	77			
CD8 TS					
High	24	15	73.3	0.384	<0.001
Low	20	72			
FOXP3					
High	31	10	53.5	0.149	0.040
Low	50	38			
Granzyme B					
High	29	35	64.1	0.276	<0.001
Low	12	55			

TE, tumor epithelium; TS, tumor stroma.

**Figure 2.**
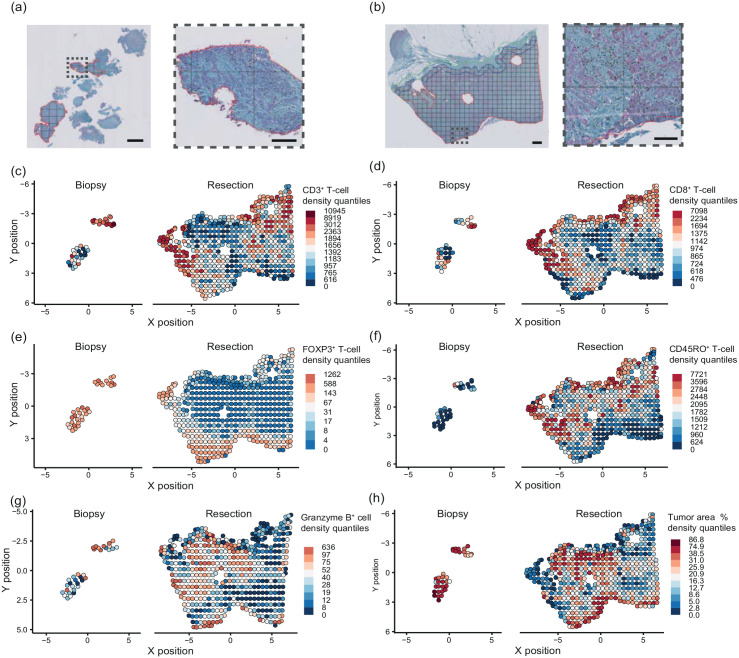
Example of tumor with discordant T-cell densities. An example of a tumor with discordant median T-cell densities is in the paired biopsy and resection sections. Screenshot of the CD8-PanCK double staining in the biopsy (a) and resection (b) sections in QuPath software. The tumor area is annotated and divided into tiles. The T-cell density in cells/mm^2^ is calculated for each tile. The scale bar represents 1 mm in the overview and 200 microns in the zoomed image. (c–h) Density heatmaps of CD3^+^ (c), CD8^+^ (d), FOXP3^+^ (e), CD45RO^+^ (f), Granzyme B^+^ (g), and T cells and tumor percentage (h) of the paired biopsy and resection sections. Each circle represents one tile. The color heatmap is scaled according to increasing 10% density quantiles.

Given the low to moderate concordances observed, we explored the possibility that intratumoral heterogeneity of T-cell densities might contribute to this phenomenon. To this end, for each tumor, the T-cell densities per tile of the whole surgical resections were plotted, and within this range, the median T-cell density of the paired biopsy was superimposed (Supplemental Figure S3). For the majority of tumors, the biopsy T-cell density deviated from the interquartile range of T-cell densities observed in the resection samples (60%, ranging per marker from 40% to 73%). For the discordant tumors, the percentage that deviated from the interquartile range was higher than 85% (ranging per marker from 68% to 97%). This implies that biopsies could not always capture the median T-cell density of the resection specimens due to intratumoral heterogeneity. An example of a discordant tumor due to spatial heterogeneity is depicted in Figure 2. There was no significant difference in analyzed biopsy tumor areas between concordant and discordant tumors, with a mean area of 2.40 mm^2^ (range 0.09–13.84) in concordant tumors versus 2.34 mm^2^ (0.04–13.99) in discordant tumors (*p* = 0.79).

**Figure 3.**
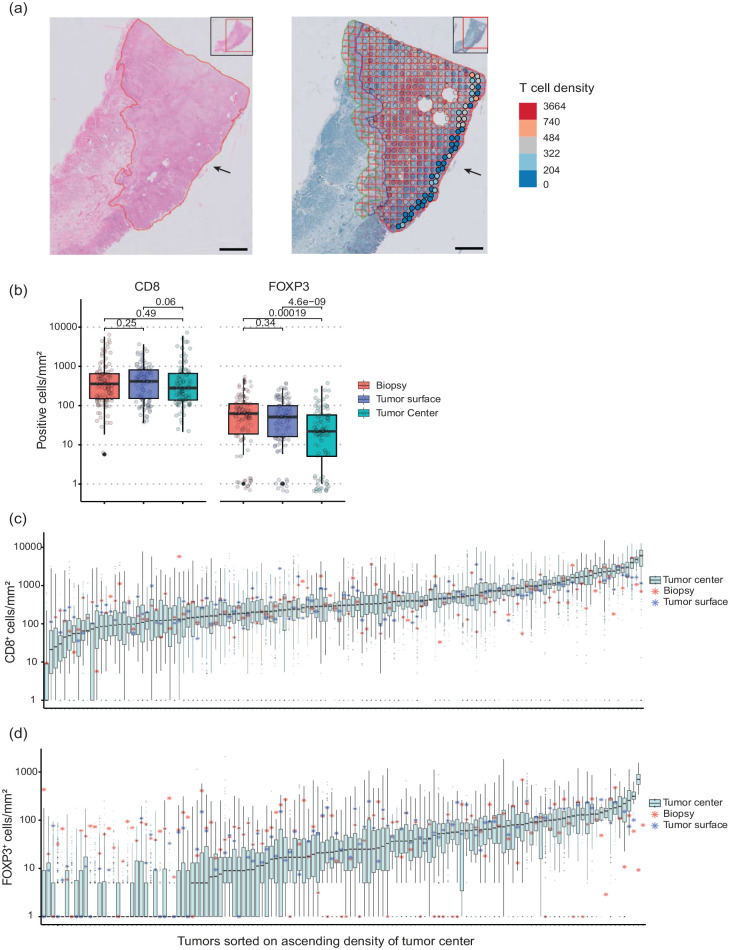
T-cell density variation in a whole section of resections with the paired biopsy and the tumor surface. (a) The tumor surface is depicted with an arrow in the H&E section of an example tumor and the CD8 IHC section of the same tumor. In the CD8 section, the T-cell density per tile was plotted in colored circles as a heatmap overlay. The non-transparent circles depicted with the black arrow were used to calculate the T-cell density of the tumor surface. The scale bar in the microphotographs depicts 2 mm. (b) The CD8^+^ and FOXP3^+^ T-cell density (cells/mm^2^) in the paired biopsy, tumor surface, and tumor center. Each circle represents one patient. (c, d) A boxplot of the T-cell densities of all tiles in each tumor section (cells/mm^2^) of the resection specimens. The samples were sorted on increasing median T-cell density. The paired biopsy median value is depicted in asterisks. The density values were offset by 1 before the log transformation. H&E, hematoxylin & eosin.

Since CD8 and FOXP3 were the most prognostic markers in the resections of the previous study, we further explored the spatial heterogeneity for these two markers in the “tumor surface” of the resection. This 1 mm tumor surface could be analyzed as a “virtual biopsy” since biopsies are sampled from the surface (Figure 3(a), the arrow depicts the mucosa: the “tumor surface”). The CD8^+^ T-cell density was comparable in biopsies compared to the tumor surface, and comparable in the tumor surface compared to the tumor center (*p* > 0.05, ns) (Figure 3(b)). The FOXP3^+^ T-cell density was comparable in biopsies compared to the tumor surface, but significantly different in the tumor surface compared to the tumor center (*p* < 0.001) (Figure 3(b)). The mean of the T-cell density in biopsies and the tumor center was plotted in the range of the tumor center in Figure 3(b) and (c). For FOXP3, the deviation of biopsy or tumor surface T-cell density from the interquartile range of the tumor center is higher than for CD8, and mostly in the tumors with the lowest T-cell densities in the resection.

**Figure 4.**
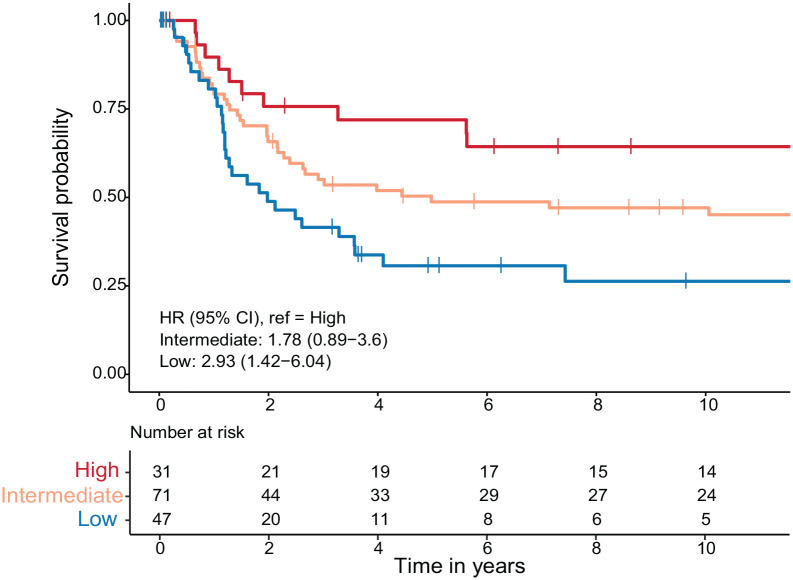
Cancer-specific survival patients stratified by biopsy Immunoscore. Kaplan–Meier analysis of cancer-specific survival with the biopsy Immunoscore as a factor. Univariable Cox regression analysis was used to calculate the HR and 95% CI of the intermediate versus high and the low versus high Immunoscore groups. CI, confidence interval; HR, hazard ratio.

Next, we explored the prognostic value of dichotomized T-cell infiltrates in the 149 biopsies, regardless of the concordance with the paired resections. CD8 and FOXP3 were significant prognostic markers in univariable Cox regression analysis, with a hazard ratio (HR; 95% confidence interval (CI)) of 1.92 (1.09–3.39) for CD8 and 1.74 (1.10–2.75) for FOXP3 (Supplemental Table 3). We devised an Immunoscore incorporating both CD8 and FOXP3, resulting in three distinct scores: a high Immunoscore indicating high levels for both markers, a low Immunoscore indicating low levels for both markers, an intermediate Immunoscore indicating one marker with high levels, and the other marker with low levels. A notable and statistically significant difference in CSS was observed between the lowest and highest Immunoscore groups; HR (95% CI): 2.93 (1.42–6.04), *p* = 0.0035 (Figure 4). To compare the Immunoscore cutoff methods in matched biopsies and resections, a similar Immunoscore was created with the CD8 and FOXP3 T-cell density in the tumor center of the resections using the cutoffs that were published previously.^7^ In the matched biopsies and resections, a statistically significant difference in CSS was observed between the lowest and highest Immunoscore groups (Supplemental Figure S4).

MSI-high and EBV^+^ tumors previously exhibited significantly higher T-cell densities in the resections and were also prognostic factors.^7^ In the paired biopsies, only EBV^+^ tumors showed significantly higher T-cell densities (Supplemental Figure S5). All 13 EBV^+^ tumors were in the Immunoscore high subgroup, whereas MSI-high tumors were distributed among all Immunoscore subgroups (*n* = 6 in high, *n* = 5 in intermediate, and *n* = 8 in low). Because of the potential association with Immunoscore, we included EBV and MSI status in a multivariable Cox regression, among other clinicopathological variables known at the time biopsies are taken. Pathological TNM stage was not included despite being a strong prognostic factor since this is determined on surgical resection specimens. The Immunoscore remained a significant independent predictor of CSS in the multivariable Cox regression that included clinicopathological variables, with an HR (95% CI) of 3.40 (1.27–9.10) for low versus high Immunoscore, *p* = 0.015 (Table 2).

**Table 2.** Multivariable Cox regression.

Variable	HR	95% CI	*p*-Value
Sex			
Female vs male	0.654	0.384–1.115	0.119
Age			
>70 vs <70	0.952	0.551–1.644	0.860
MSI-status			
MSI-high vs MSS	1.618	0.787–3.330	0.191
EBV-status			
EBV^+^ vs EBV^–^	1.666	0.455–6.101	0.441
Lauren subtype			
Diffuse/mixed vs intestinal	1.566	0.862–2.846	0.141
Other vs intestinal	2.109	1.049–4.242	0.036
Immunoscore CD8-FOXP3			
Low vs high	3.402	1.273–9.095	0.0147
Intermediate vs high	2.280	0.868–5.989	0.0945

CI, confidence interval; EBV^+^, Epstein-Barr virus positive; EBV^–^, Epstein-Barr virus negative; HR, hazard ratio; MSI, microsatellite instability; MSS, microsatellite stable.

## Discussion

Our findings demonstrate a low to moderate concordance for different T cells between biopsies and resections. This implicates that biopsies are not fully representative fragments of the entire tumor and its immune microenvironment, particularly considering previous evidence that immune cells in the invasive margin, which is not reached with an endoscopic biopsy, possess a more robust prognostic value in GC compared to those found in the tumor center.^7,11,24,25^ This makes it unclear whether GC biopsy-based T-cell densities could become a reliable prognostic marker. Despite the limited concordance, our study however revealed a significant association between a biopsy-based Immunoscore, incorporating both CD8 and FOXP3, and improved prognosis. High cytotoxic CD8^+^ T-cell densities were previously associated with improved prognosis in GC.^26^ The role of FOXP3, an immunosuppressive marker, is not well understood since associations with poor as well as with improved prognosis were reported in treatment-naïve GC patients.^6,26,27^ Possibly, FOXP3^+^ T-cell infiltration reflects the inflamed “hot” immune state of the tumor, where immune suppressive cells were later recruited as part of an immune evasive response of the tumor.^28^ Indeed, in our study, in the resection specimens of the same patients FOXP3 and CD8 T-cell densities were positively correlated and exhibited the most robust prognostic value as well.^7^ Regardless of the suboptimal concordance of these markers between biopsies and resection specimens, CD8 and FOXP3 still retain prognostic significance in small biopsies. This finding supports the use of a biopsy-based Immunoscore as a valuable prognostic biomarker.

The spatial heterogeneity of immunohistochemical biomarkers was also reported for those currently used for GC: HER2^29–31^ and PD-L1.^32,33^ Since HER2-targeted therapy^34^ and anti-PD-1/PD-L1^35,36^ are recommended for patients with unresectable, advanced gastroesophageal cancer, biopsies are often the only available tissue, and therefore studies have assessed the concordance with the paired resections. HER2 showed a concordance rate of 70%–95% between biopsy and resection specimens.^37–40^ For PD-L1, the reported concordance rates varied between 63% and 100%.^41–43^ The concordance rate of CD8 was highest in our study and with 85% comparable to PD-L1 and HER2. Therefore, CD8 could be the most reliable and promising biopsy-based biomarker.

Since the concordance of CD8 in biopsies and resections is highest, it is an attractive potential biomarker for the prediction of response to neoadjuvant therapy. It is hypothesized that neoadjuvant chemo(radio)therapy and/or immunotherapy could benefit patients most by activating the immune microenvironment while the tumor bulk is still present, thereby targeting tumor antigens, which could contribute to a long-term immunological effect following resection.^10,44,45^ CD8^+^ T cells were associated with benefit from immune checkpoint inhibitors (ICI) in multiple tumors and treatment combinations in a systematic review and meta-analysis.^46^ Specifically, in pre-treatment samples, high CD8^+^ T-cell densities have demonstrated favorable responses to immunotherapy in melanoma^47^ and early-stage mismatch repair proficient colon cancer.^48^ In GC, tumors with high PD-L1 expression, MSI, and EBV are considered as most responsive to immunotherapy. Since all of these markers are associated with high CD8^+^ T-cell densities, CD8 could serve as a potential biomarker for immunotherapy response.^49,50^ Immunotherapy is continuously advancing in GC, with multiple trials investigating (neo)adjuvant ICI combinations in non-metastatic disease.^51–53^ In the phase II PANDA trial, neoadjuvant atezolizumab (anti-PD-L1) and subsequent chemotherapy led to a major pathological response.^52^ CD8^+^ T cells were increased in biopsies of responders after atezolizumab monotherapy. Transcriptomic data supported this finding with immune-related gene expression in on-treatment biopsies. Since the gene expression levels were comparable in post-treatment surgical resections of these patients, the effects on CD8 expression were considered treatment-related. Furthermore, CD8^+^PD1^+^ T cells were predictive of treatment response.^52^ In another study, CD8^+^PD-1^+^LAG3^−^ T cells assessed on pre-treatment surgical resections were also predictive of ICI response in GC.^54^ Given these findings, it is pertinent to further investigate whether CD8^+^ T-cell populations in biopsy specimens could serve as predictive markers for response to neoadjuvant immunotherapy in GC, potentially in combination with PD-1 and PD-L1 assessment.^55^

FOXP3 displayed the lowest concordance, consistent with a comparative study in colon cancer.^56^ FOXP3^+^ regulatory T cells are present at much lower densities, and therefore more prone to sampling error due to spatial heterogeneity. This was reflected by the large range of T-cell densities within one tumor, especially in the tumors with the lowest T-cell densities in the resection. Indeed, for those tumors with the lowest median densities, the discordance between biopsy and resection was more frequent.

However, there was a tendency toward higher FOXP3 T-cell densities in biopsies and sampling error should result in discordance in both directions. This could suggest a different expression in the superficial part of the tumor where endoscopic biopsies are taken. Differences between the superficial and deeper part of the tumor have been previously demonstrated concerning RNA expression and genomic abberations^57^ and HER2 protein expression.^58^ At the surface of the tumor, there is also inflammation present due to mucosal damage. Regulatory T cells could possibly be associated with this non-tumor-related inflammation, and therefore more expressed at the tumor surface. Cheng et al.^59^ showed that the number of FOXP3^+^ T cells was increased in gastric biopsies of patients with chronic gastritis, peptic ulcer, and GC compared to healthy controls. The number of FOXP3^+^ T cells was also correlated with acute inflammation, chronic inflammation, and Helicobacter pylori infection. Regulatory T cells present at such precursor lesions^60^ possibly contribute to the development of GC by suppressing the antitumor response.^61^ These pre-cursor lesions and chronic inflammation are more frequently associated with intestinal-type tumors, which may explain a difference in discordance between the intestinal- and diffuse-type tumors.

Despite the challenge of heterogeneity in biomarker expression, studies showed that sampling multiple biopsies could improve performance. A minimum of four to five endoscopic biopsies achieved a good concordance for both HER2^37,40,58^ and PD-L1.^62^ This recommendation aligns with the ESMO guidelines, which suggest that obtaining five to eight endoscopic biopsies can provide a sufficient representation of the tumor.^63^ The average of tumor-containing biopsies in this study was 3, which is an important limitation. Future studies should evaluate whether analysis of an increased number of biopsies could also improve the concordance and the prognostic value of a biopsy-based Immunoscore.

This is the first study that compares T-cell infiltrates in paired diagnostic biopsies and resections in patients who did not receive neoadjuvant treatment. This study was performed to explore the suitability of T cells as a biopsy-based biomarker and is still far from clinical implementation. Validation of the prognostic value of the Immunoscore is necessary. A limitation of this study is that the Immunoscore was not assessed in patients who received the standard of care, which is perioperative chemotherapy. Yet, our results can provide awareness of the impact of spatial heterogeneity of T cells in studies where pre-treatment biopsies are compared to post-treatment resections. Changes in T-cell densities are often attributed to the effect of the chemotherapy treatment, without recognizing sampling error due to heterogeneity or reporting the number of analyzed biopsies.^64–67^ Future studies should acknowledge the influence of spatial heterogeneity, potentially by an additional comparison of T cells measured in post-treatment resections with treatment-naïve resections.

## Conclusion

Despite a moderate concordance of T-cell densities between biopsy and resection specimens, a biopsy-based Immunoscore could identify distinct subgroups with a promising prognostic value. To fully evaluate the prognostic performance of an Immunoscore as well as its value in response prediction, additional studies are warranted. CD8 was the most promising T-cell marker with the highest concordance between biopsy and resections and should be included in future studies.

## Supplemental Material

sj-docx-1-tam-10.1177_17588359241287747 – Supplemental material for A biopsy-based Immunoscore in patients with treatment-naïve resectable gastric cancer Supplemental material, sj-docx-1-tam-10.1177_17588359241287747 for A biopsy-based Immunoscore in patients with treatment-naïve resectable gastric cancer by Tanya T. D. Soeratram, Isis Beentjes, Jacqueline M. P. Egthuijsen, Aart Mookhoek, Marilyne M. Lange, Elma Meershoek-Klein Kranenbarg, Henk H. Hartgrink, Cornelis J. H. van de Velde, Bauke Ylstra, Hanneke W. M. van Laarhoven and Nicole C. T. van Grieken in Therapeutic Advances in Medical Oncology

sj-docx-2-tam-10.1177_17588359241287747 – Supplemental material for A biopsy-based Immunoscore in patients with treatment-naïve resectable gastric cancer Supplemental material, sj-docx-2-tam-10.1177_17588359241287747 for A biopsy-based Immunoscore in patients with treatment-naïve resectable gastric cancer by Tanya T. D. Soeratram, Isis Beentjes, Jacqueline M. P. Egthuijsen, Aart Mookhoek, Marilyne M. Lange, Elma Meershoek-Klein Kranenbarg, Henk H. Hartgrink, Cornelis J. H. van de Velde, Bauke Ylstra, Hanneke W. M. van Laarhoven and Nicole C. T. van Grieken in Therapeutic Advances in Medical Oncology

## Declarations

**Ethics approval and consent to participate:** This study was in accordance with the Declaration of Helsinki. The D1/D2 trial was approved by the medical ethical committee of the Leiden University Medical Center. The Dutch Code of Conduct for Responsible Use of Human Tissue allows for the analysis of residual tissue specimens obtained for diagnostic purposes and anonymized publication of the study results. The biobank committee of Amsterdam UMC (number BUP2016-71) and the nationwide network and registry of histo- and cytopathology in the Netherlands (PALGA: Dutch Pathology Registry, number LZV 2018-122) have approved to use of archival tumor material for this study. Verbal consent was obtained from the participants of the D1/D2 trial.

**Consent for publication:** Verbal consent was obtained from the participants of the D1/D2 trial.

**Availability of data and materials:** Data are available from the corresponding author upon reasonable request.

**Disclaimer:** Hanneke van Laarhoven is an Associate Editor of Therapeutic Advances in Medical Oncology and an author of this paper; therefore, the peer-review process was managed by alternative members of the board and the submitting editor has not been involved in the decision-making process.
