# Supplementary material for: A biopsy-based Immunoscore in patients with treatment-naïve resectable gastric cancer
Source: Ther Adv Med Oncol. 2024 Oct 21;16:17588359241287747. doi: 10.1177/17588359241287747 (PMC11497501; doi:10.1177/17588359241287747)
Supplement: sj-docx-1-tam-10.1177_17588359241287747 – Supplemental material for A biopsy-based Immunoscore in patients with treatment-naïve resectable gastric cancer [file sj-docx-1-tam-10.1177_17588359241287747.docx]

**Title:** A biopsy-based immunoscore in patients with treatment-naïve resectable gastric cancer

**Authors and full affiliations**

Tanya T.D. Soeratram, MSC^1,2^; Isis Beentjes, BSc^1,2^; Jacqueline M.P. Egthuijsen, BSc^1,2^; Aart Mookhoek, MD, PhD^4^; Marilyne M. Lange, MD, PhD^1^; Elma Meershoek-Klein Kranenbarg, MSc^3^; Henk H. Hartgrink, MD, PhD^3^; Cornelis J.H. van de Velde, MD, PhD^3^; Bauke Ylstra, PhD^1,2^; Hanneke W.M. van Laarhoven, MD, PhD, PhD^2,5^; Nicole C.T. van Grieken, MD, PhD^1,2*^

^1^Department of Pathology, Amsterdam UMC location Vrije Universiteit Amsterdam, Amsterdam, The Netherlands ^2^Cancer Center Amsterdam, Cancer Biology and Immunology, Amsterdam, The Netherlands
^3^Department of Surgery, Leiden University Medical Center, Leiden, The Netherlands
^4^Institute of Tissue Medicine and Pathology, University of Bern, Bern, Switzerland
^5^Department of Medical Oncology, Amsterdam UMC location University of Amsterdam, Amsterdam, The Netherlands

*Corresponding author
Prof. Dr. Nicole van Grieken
Amsterdam UMC location Vrije Universiteit Amsterdam, Pathology, De Boelelaan 1117, Amsterdam, The Netherlands

**Supplementary Materials - Index**

| **Supplementary Tables** |  |
| --- | --- |
| Supplementary Table 1 | *pag. 3* |
| Supplementary Table 2 | *pag. 4* |
| Supplementary Table 3 | *pag. 5* |
| **Supplementary Figures** |  |
| Supplementary Figure S1 | *pag. 6* |
| Supplementary Figure S2 | *pag. 7* |
| Supplementary Figure S3 | *pag. 8-9* |
| Supplementary Figure S4 | *pag. 10* |
|  |  |

**Supplementary Tables**

**Supplementary Table 1. Baseline characteristics of patients included in the study**

|  | | All patients with biopsy (*n*=149) | | Matched biopsy-resection pairs only (*n*=131) | |
| --- | --- | --- | --- | --- | --- |
|  | | *n* | % | *n* | % |
| EBV status | |  |  |  |  |
|  | EBV+ | 13 | 9% | 13 | 10% |
|  | EBV- | 135 | 91% | 118 | 90% |
|  | Missing | 1 | 1% |  |  |
| MSI-status | |  |  |  |  |
|  | MSI-high | 19 | 13% | 15 | 11% |
|  | MSS | 127 | 85% | 116 | 89% |
|  | Missing | 3 | 2% |  |  |
| Sex |  |  |  |  |  |
|  | Male | 77 | 52% | 72 | 55% |
|  | Female | 72 | 48% | 59 | 45% |
| Age |  |  |  |  |  |
|  | >70 | 48 | 32% | 41 | 31% |
|  | ≤70 | 101 | 68% | 90 | 69% |
| Lauren classification  (determined on biopsy) | | |  |  |  |
|  | Intestinal | 81 | 54% | 73 | 56% |
|  | Diffuse | 46 | 31% | 37 | 28% |
|  | Mixed | 5 | 3% | 5 | 4% |
|  | Other | 17 | 11% | 16 | 12% |
| pT stage | |  |  |  |  |
|  | T1-2 | 46 | 31% | 34 | 26% |
|  | T3-4 | 103 | 69% | 97 | 74% |
| pN stage | |  |  |  |  |
|  | N0 | 60 | 40% | 51 | 39% |
|  | N1-3 | 89 | 60% | 80 | 61% |

EBV+, Epstein-barr virus positive; EBV-, Epstein-barr virus negative; MSI, microsatellite instability; MSS, microsatellite stable; pT stage, pathological T stage; pN stage, pathological N stage

**Supplementary Table 2. Concordance of biopsies and resections in Lauren’s histological subtypes**

|  | Intestinal type | | | Diffuse/mixed type | | |
| --- | --- | --- | --- | --- | --- | --- |
|  | Percent concordant | Cohen's κ | *P*-value | Percent concordant | Cohen's κ | *P*-value |
| CD3 | 58.9 | 0.188 | 0.090 | 45.2 | -0.136 | 0.188 |
| CD45RO | 68.5 | 0.242 | 0.039 | 78.6 | 0.270 | 0.079 |
| CD8 | 86.3 | 0.676 | 0.000 | 78.6 | 0.276 | 0.065 |
| CD8 TE | 80.8 | 0.587 | 0.000 | 61.9 | 0.109 | 0.343 |
| CD8 TS | 72.6 | 0.428 | 0.000 | 66.7 | 0.148 | 0.323 |
| FOXP3 | 49.3 | 0.075 | 0.446 | 59.5 | 0.261 | 0.040 |
| Granzyme B | 64.4 | 0.313 | 0.004 | 57.1 | -0.005 | 0.969 |

TE, tumor epithelium; TS, tumor stroma

**Supplementary Table 3. Univariable Cox regression**

| Variable |  | HR | 95% CI | *P*-value |
| --- | --- | --- | --- | --- |
| CD3 | Low vs High | 1.546 | 0.889-2.688 | 0.123 |
| CD8 | Low vs High | 1.923 | 1.091-3.389 | 0.024 |
| CD8 TE | Low vs High | 1.478 | 0.914-2.390 | 0.111 |
| CD8 TS | Low vs High | 1.782 | 1.058-3.001 | 0.030 |
| CD45RO | Low vs High | 1.685 | 0.926-3.068 | 0.088 |
| FOXP3 | Low vs High | 1.737 | 1.099-2.746 | 0.018 |
| Granzyme B | Low vs High | 0.709 | 0.434-1.160 | 0.171 |
| Sex | Female vs Male | 0.717 | 0.454-1.132 | 0.153 |
| MSI status | MSI-high vs MSS | 1.085 | 0.572-2.058 | 0.804 |
| EBV status | EBV+ vs EBV- | 0.756 | 0.305-1.873 | 0.545 |
| Age | >70 vs <70 | 0.941 | 0.572-1.546 | 0.809 |
| Lauren classification | Diffuse/mixed vs Intestinal | 1.346 | 0.816-2.220 | 0.245 |
|  | Other vs Intestinal | 2.915 | 1.527-5.564 | 0.002 |

TE, tumor epithelium; TS, tumor stroma; EBV+, Epstein-barr virus positive; EBV-, Epstein-barr virus negative; MSI, microsatellite instability; MSS, microsatellite stable

**Supplementary Figures**


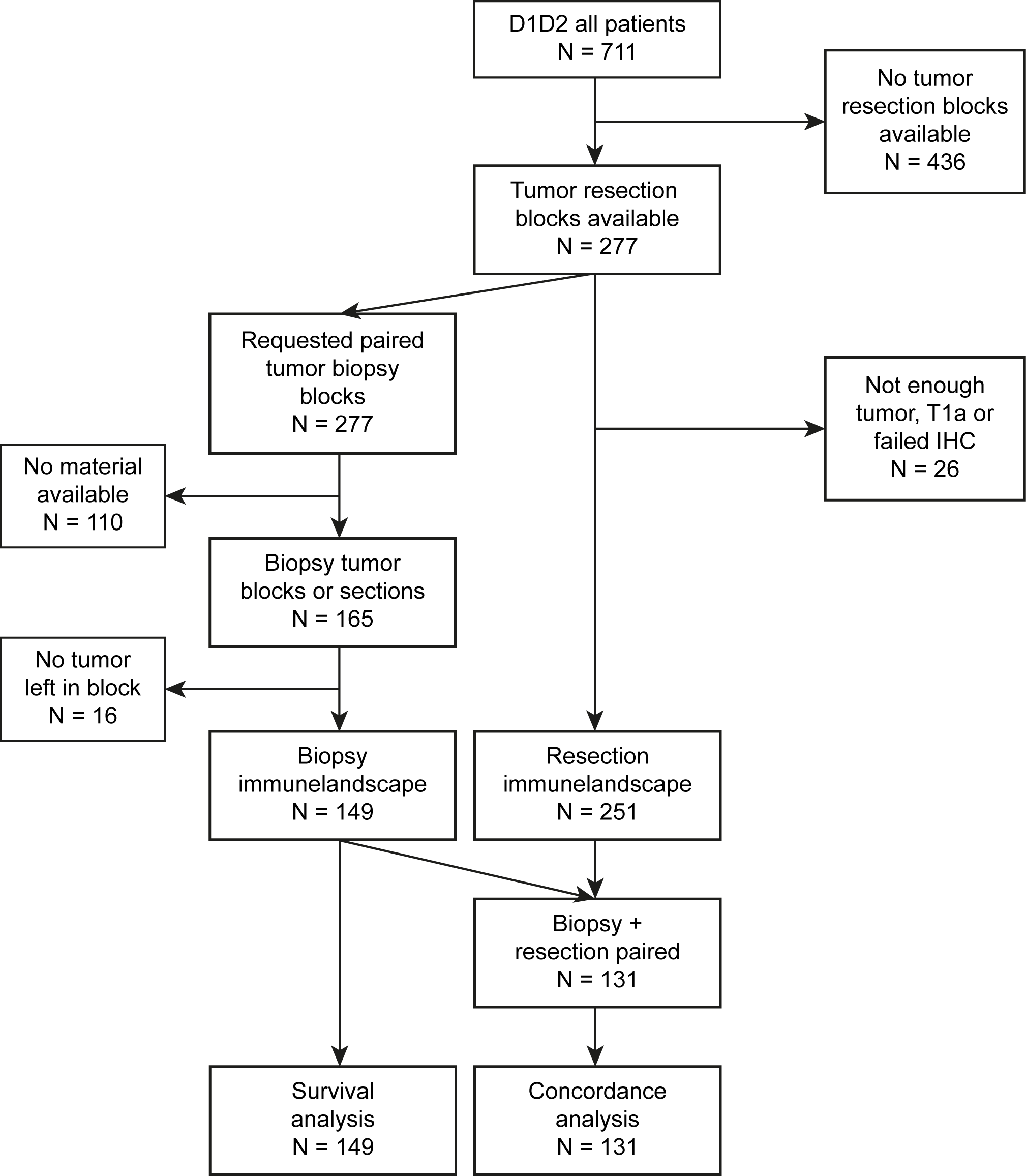


**Supplementary Figure S1. Flowchart of tumor samples included in the study**

**
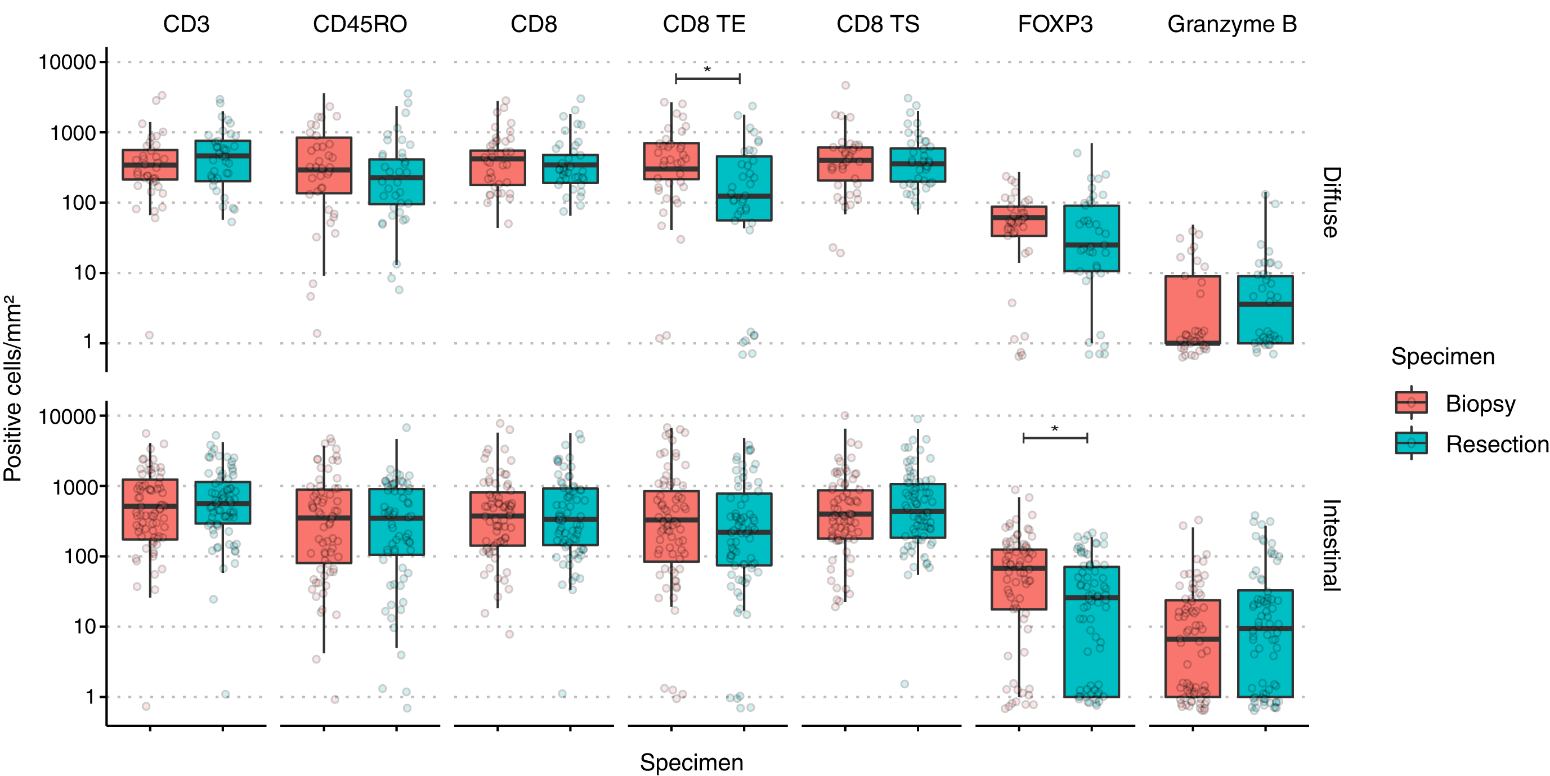
Supplementary Figure S2. Median T-cell densities in paired biopsy and resection specimens in Lauren’s diffuse and intestinal type**The median T-cell density value of all tiles in the tumor section (cells/mm^2^) of the biopsy and resection specimens in the diffuse and intestinal type of Lauren’s histological classification. Each circle represents one tumor sample. The boxplots depict the median and interquartile range of all tumor samples. The density values were offset by 1 before log transformation. The statistical difference between biopsy and resection was determined by the Wilcoxon ranked paired sum test before log-transformation. TE, tumor epithelium; TS, tumor stroma

**
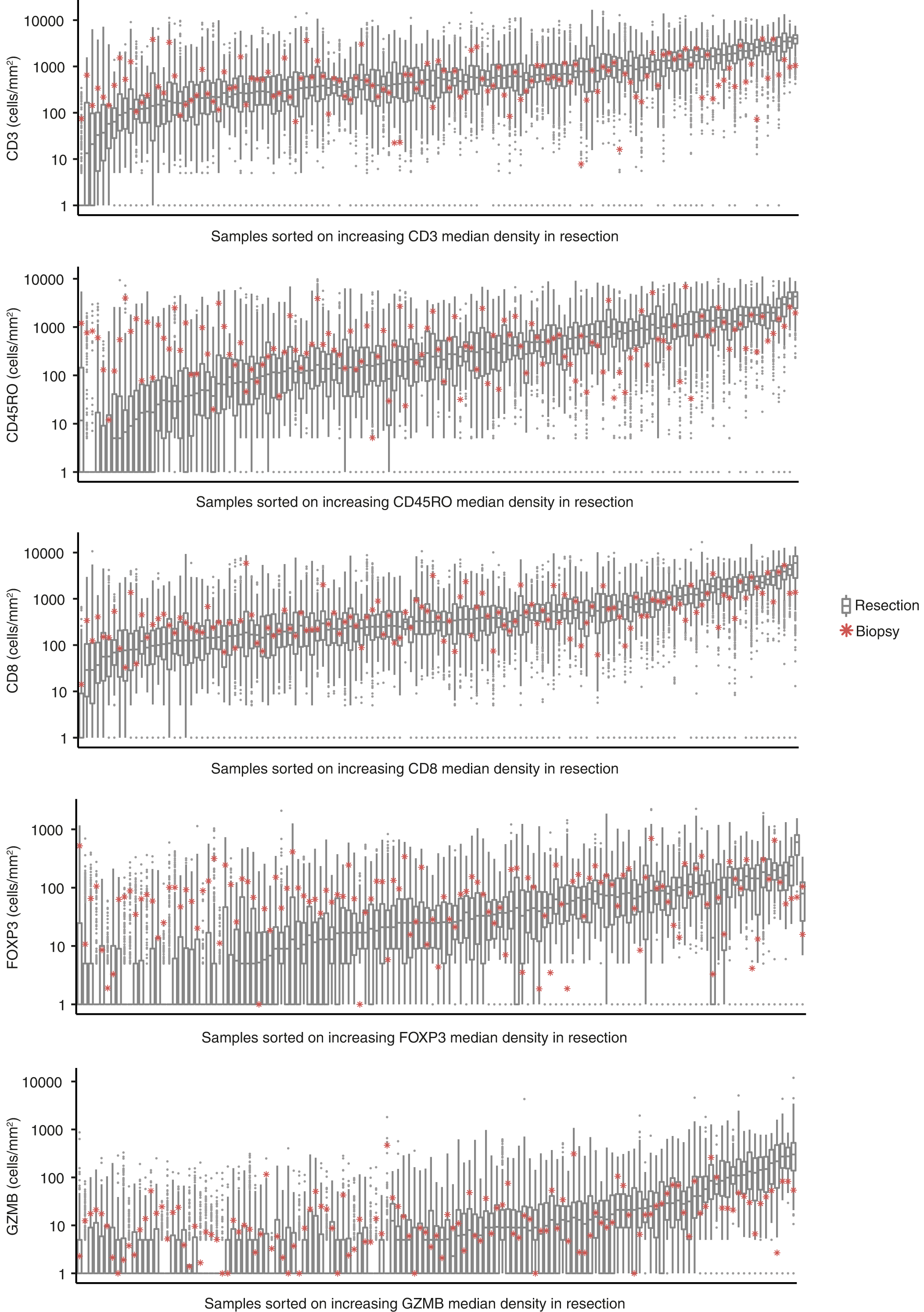
Supplementary Figure S3. T-cell density variation in whole section of resections with paired biopsy**A boxplot of the T-cell densities of all tiles in each tumor section (cells/mm^2^) of the resection specimens. The samples were sorted on increasing median T-cell density. The paired biopsy median value is depicted in asterisks. The density values were offset by 1 before log transformation.

**
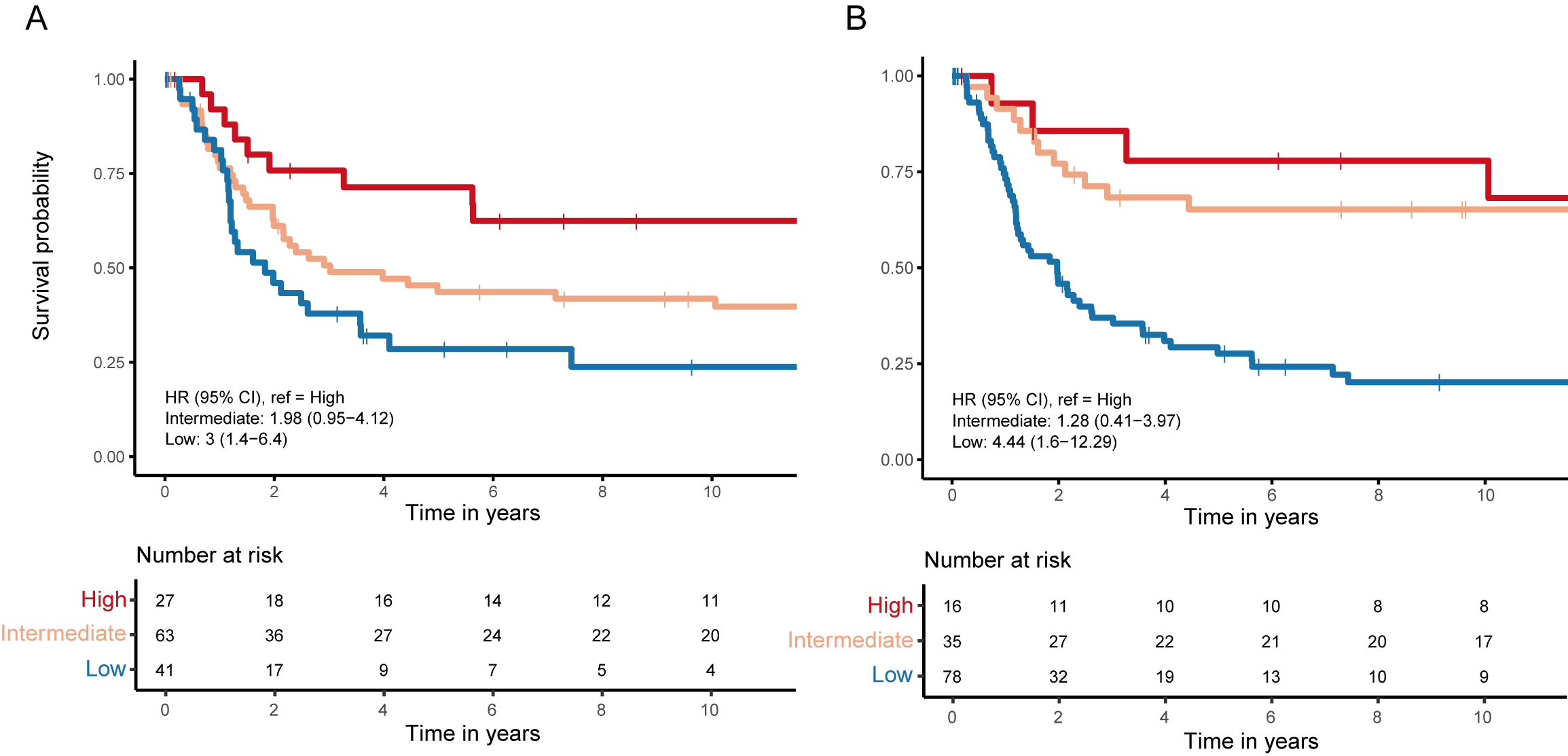
 Supplementary Figure S4. Cancer-specific survival patients stratified by immunoscore in matched biopsies and resections**

Kaplan-Meier analysis of cancer-specific survival with the CD8-FOXP3 immunoscore as factor. Univariable Cox regression analysis was used to calculate the hazard ratio (HR) and 95% confidence intervals (CI) of the intermediate vs high and the low vs high immunoscore groups in 131 matched (A) biopsies and (B) resections.


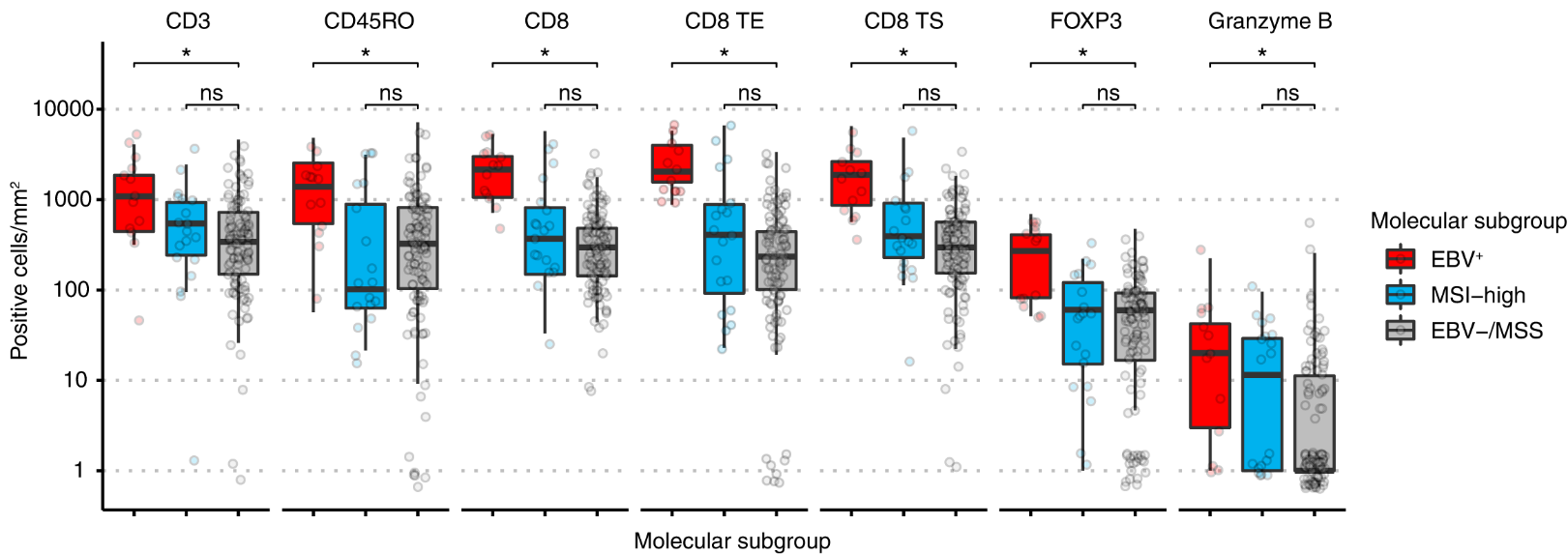


**Supplementary Figure S5.** **Median T-cell densities in biopsies in EBV-positive and MSI-high tumors**

The median T-cell density value of all tiles in the tumor section (cells/mm^2^) of the biopsy in EBV-positive (EBV^+^), microsatellite instability high (MSI-high) compared to EBV-negative and microsatellite stable (EBV-/MSS) tumors. Each circle represents one tumor sample. The boxplots depict the median and interquartile range of all tumor samples. The density values were offset by 1 before log transformation. The statistical difference was determined by the Wilcoxon test before log-transformation. TE, tumor epithelium; TS, tumor stroma.
